# Heterozygous versus homozygous phenotype caused by the same *MC4R* mutation: novel mutation affecting a large consanguineous kindred

**DOI:** 10.1186/s12881-018-0654-1

**Published:** 2018-08-02

**Authors:** Max Drabkin, Ohad S. Birk, Ruth Birk

**Affiliations:** 1 The Morris Kahn Laboratory of Human Genetics at the National Institute of Biotechnology in the Negev, Ben-Gurion University of the Negev, Beer-Sheva, Israel; 2 Genetics Institute, Soroka University Medical Center, Faculty of Health Sciences, Ben-Gurion University of the Negev, Beer-Sheva, Israel; 3 Department of Nutrition, Faculty of Health Sciences, Ariel University, Ariel, Israel

**Keywords:** MC4R, Obesity, Mutation, Homozygous, Heterozygous

## Abstract

**Background:**

The hypothalamic G-protein-coupled-receptor melanocortin-4 receptor (MC4R) is a key player in the central circuit regulating energy expenditure and appetite. Heterozygous loss-of-function *MC4R* mutations are the most common known genetic cause of monogenic human obesity, with more than 200 mutations described to date, affecting 2–3% of the population in various cohorts tested. Homozygous or compound heterozygous *MC4R* mutations are much less frequent, and only few families have been described in which heterozygotes and homozygotes of the same mutation are found.

**Methods:**

We performed exome sequencing in a consanguineous Bedouin family with morbid obesity to identify the genetic cause of the disease. Clinical examination and biochemical assays were done to delineate the phenotype.

**Results:**

We report the frequency of *MC4R* mutations in the large inbred Bedouin Israeli population. Furthermore, we describe consanguineous inbred Bedouin kindred with multiple individuals that are either homozygous or heterozygous carries of the same novel *MC4R* mutation (c.124G > T, p.E42*). All family members with the homozygous mutation exhibited morbid early-onset obesity, while heterozygote individuals had either a milder overweight phenotype or no discernable phenotype compared to wild type family members. While elder individuals homozygous or heterozygous for the *MC4R* mutation had abnormally high triglycerides, cholesterol, glucose and HbA1C levels, most did not.

**Conclusions:**

*MC4R* mutation homozygotes exhibited morbid early-onset obesity, while heterozygotes had a significantly milder overweight phenotype. Whereas obesity due to *MC4R* mutations is evident as of early age – most notably in homozygotes, the metabolic consequences emerge only later in life.

## Background

Obesity, affecting more than 30% of adults and children worldwide [1], is a complex trait affected by diet in conjunction with environmental and genetic factors [2]. The melanocortin-4 receptor (MC4R) is a key player in the leptin-regulated melanocortin circuit, essential for central energy regulation [3]. Predominantly expressed in the hypothalamus, the G-protein-coupled-receptor MC4R acts in signaling satiety, consequently decreasing food intake: upon binding of its endogenous ligand, the neuropeptide melanocyte stimulating hormone (α-MSH), MC4R activates adenylate cyclase, enhancing cAMP synthesis and levels, thereby generating a satiety signal. In line with the role of MC4R in satiety signaling and regulation of food intake, MC4R null mutant (MC4R ^−/−^) mice develop severe obesity, while heterozygous (MC4R ^+/−^) mice present a mildly obese intermediate phenotype [4].

Mutations in members of the leptin-regulated melanocortin circuit have been shown to result in human obesity. Most notably, human obesity has been associated with mutations in leptin (LEP) [5], leptin receptor (LEPR) [6], prohormone convertase 1 (PC1) [7], proopiomelanocortin (POMC) [8] and melanocortin-4 receptor (MC4R) [9–15]. In fact, heterozygous loss-of-function *MC4R* mutations have been shown to be the most common known genetic cause of human obesity. Initial studies by Farooqi et al. [16] estimated that about 6% of early onset morbidly obese patients harbor *MC4R* mutations. Consequently, other large cohort studies, mostly in Caucasian European and American populations, demonstrated lower calculated prevalence of about < 2% [17–21]. The prevalence of *MC4R* mutations in severely obese populations in other ethnicities, such as Asians, was found to be low or not relevant [16].

Homozygous or compound heterozygous carriers of *MC4R* mutations are rare [15, 16, 22, 23]. Previous studies suggest that the obesity in these rare cases develops earlier in life, and is more severe than for heterozygous carriers; notably, these homozygous individuals do not display any discernible additional unrelated phenotypes [16]. Only few families have been described to date in which multiple heterozygotes and homozygotes of the same mutation are found [16]. We now describe large consanguineous inbred kindred with individuals that are either homozygous or heterozygous carries of the same novel *MC4R* mutation, and review the literature of such families described to date.

## Methods

### Subjects and clinical phenotyping

**Fig. 1:**
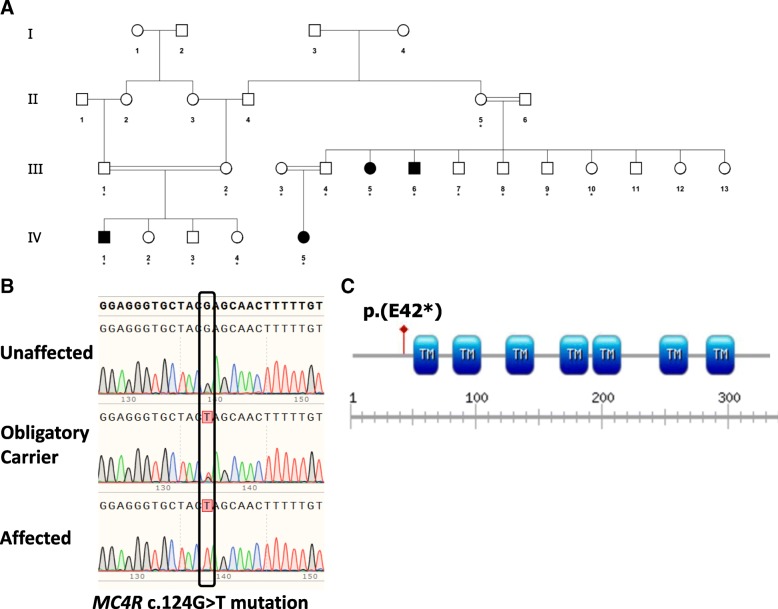
Pedigree of the studied kindred and the *MC4R* mutation. **a** Pedigree of a consanguineous Bedouin family presenting with a phenotype of autosomal recessive early-onset obesity (individuals with phenotypic morbid obesity are marked as affected; asterisk marks individuals whose DNA was available for analysis). **b** The c.124 G > T, p.(E42*) *MC4R* mutation: Sanger sequencing of an unaffected individual (III:7), an obligatory carrier (II:5) and a morbidly-obese affected individual (IV:1). **c** Schematic representation of the p.(E42*) mutation in *MC4R* predicted to truncate almost 90% of the protein (TM, Transmembrane domain)

Sixteen affected and unaffected individuals of consanguineous Bedouin kindred were studied (Fig. 1a). DNA samples were obtained following informed consent and approval of the Soroka Medical Center Internal Review Board (0316–14-SOR) according to Helsinki ethical guidelines. Clinical phenotyping was determined by an experienced team of pediatrics specialists and clinical geneticists for all affected individuals, their parents and siblings. Blood samples, as well as measurements of weight and height, were taken from all participants on the same day.

### Whole exome sequencing

Genomic DNA was isolated from peripheral blood using The E.Z.N.A. SQ Blood DNA Kit (Omega Bio-tek, Norcross,GA, USA). Whole exome sequencing (HiSeq2000, Illumina, San Diego, CA, USA) of two affected individuals (III:5 & IV:1, Fig. 1a) was performed using paired-end (2 × 100) protocol at a mean coverage of 100-fold (85–90% of all exonic nucleotides were covered by > 100 reads), as previously described [24]. For exome enrichment, we used NimbleGen SeqCap EZ Human Exome Library v2.0 (Roche NimbleGen, Madison, WI, USA) targeting 44.1 Mb regions. Sequencing read alignment, variant calling and annotation were performed by DNAnexus (DNAnexus Inc., Mountain View, CA, USA; dnanexus.com).

Data were analyzed using QIAGEN’s Ingenuity® Variant Analysis™ software (www.qiagen.com/ingenuity) from QIAGEN Redwood City. Using their filtering cascade, we excluded common variants which demonstrated an allele frequency higher than 0.5% in AFC (Allele Frequency Community - including ExAC and CGI), in the 1000 genomes project and in NHLBI ESP exomes (National Heart, Lung, and Blood Institute Exome Sequencing Project). In addition, we excluded variants which appeared in a homozygous state or that presented an allele frequency of higher than 2% in our in-house whole exome sequencing database of 120 Bedouin control samples. Furthermore, we kept variants which were predicted to have a deleterious effect upon protein coding sequences (e.g. Frameshift, in-frame indel, stop codon change, missense or predicted to disrupt splicing by MaxEnt Scan) and variants which were experimentally observed to be associated with a phenotype: pathogenic, possibly pathogenic or disease-associated, according to the Human Gene Mutation Database (HGMD). Following the above filtering, of the remaining variants we selected only homozygous variants that were shared between both affected individuals sequenced.

### Mutation screening

Validation of the *MC4R* variant in all affected family members was performed by Sanger sequencing. Primers used for PCR: Forward 5’-ATCAATTCAGGGGGACACTG; Reverse 5’-AACGCTCACCAGCATATCAG. Annealing temperature used was 60 °C and the extension time was set for 30 s. Segregation analysis within the entire kindred was performed by restriction analysis using the primers mentioned above for PCR, based on an NheI restriction site generated through the *MC4R* c.124 G > T mutation. PCR products (207 bp amplicon) were incubated for 3 h with the enzyme NheI (New England Biolabs) which cuts selectively only the mutant allele (163 and 44 bp fragments), and subsequently loaded onto a 2% agarose gel for electrophoresis.

## Results

### Molecular genetic studies

In search for the molecular basis of the familial obesity, whole-exome sequencing analysis was done for two remotely related affected individuals of the kindred, namely III:5 & IV:1 (Fig. 1a). Data were filtered for normal variants as described in the materials and methods section. Following the above filtering, only a single homozygous variant was found that was shared between the two individuals: g.chr18: 58039459C < A, c.124 G > T, p.E42* in *MC4R*. No other variants (heterozygous or homozygous) in *MC4R* or in any other obesity-related gene were identified in any of the two subjects tested through whole exome sequencing. The *MC4R* mutation, validated by Sanger sequencing (Fig. 1b), was found to segregate within the family as expected for autosomal recessive heredity (data not shown). There is no evidence of the reported mutation being present in the Genome Aggregation Database (gnomAD, http://gnomad.broadinstitute.org/), a database of 123,136 exome sequences and 15,496 whole-genome sequences from unrelated individuals sequenced as part of various disease-specific and population genetic studies. The mutation truncates the 332-amino acid MC4R protein at amino acid 42, eliminating all its 7 transmembrane domains, hence practically all its functional domains (Fig. 1c). The mutation was not found in 120 whole exome sequences of local Bedouin controls in our in-house database.

As delineated in Table 1, of those 120 Bedouin control non-related individuals, one carried a heterozygous c.606C > A p.F202 L *MC4R* mutation previously associated with obesity, and two individuals carried heterozygous synonymous variants (c.594C > T, c.690C > T) previously described as possibly associated with obesity, though likely non-pathogenic [25–27]. Clinical obesity-related information regarding those individuals is not available. Interestingly, 4 of the 120 Bedouin controls were heterozygous for the c.307G > A p.V103I *MC4R* variant previously associated with protection from obesity [28] (ExAC frequency for this variant is 0.01743).

**Table 1:** *MC4R* variants identified in 120 ethnically matched controls, excluding sequenced members of the studied kindred

Mutation Type	Transcript Variant	Protein Variant	No. of subjects with genotype (All heterozygous)	dbSNP ID	ExAC Frequency
Synonymous	c.594C > T	p.I198I	1	61,741,819	0.003676
Synonymous	c.690C > T	p.P230P	1	148,026,669	2.472e-05
Missense	c.606C > A	p.F202 L	1	138,281,308	0.0008488
Missense	c.307G > A	p.V103I	4	2,229,616	0.01743
Stop gain	c.124G > T	p.E42	0	–	–

### Clinical characterization

Sixteen individuals of a consanguineous Bedouin kindred were studied (ages 1–58 years, 21.7 ± 14.7 SD), of which 4 were homozygous for the *MC4R* mutation, 7 were heterozygous for the mutation and 5 were homozygous for the wild type sequence. As shown in Table 2, height, weight and body mass index (BMI) were measured for all, and fasting blood values of cholesterol, triglycerides, LDL, HDL, glucose and HbA1C were available for most. In cases where more than one measurement was available, the average of the measurements was calculated and is presented.

**Table 2:** Phenotypic delineation of the 16 family members studied

ID	Sex	Genotype	Age (years)	BMI	Cholesterol (mg/dl)	LDL (mg%)	HDL (mg/dl)	TG (mg/dl)	Glucose (mg/dl)	HbA1C (%)
III:5	F	Hom	25–30	48	358	N/A	63	444	413	16
III:6	M	Hom	25–30	57	156	107	36	66	86	6.2
IV:1	M	Hom	5–10	36 (3.1)	153	93	28	162	73	5.5
IV:5	F	Hom	5–10	37 (3.1)	150	80	37	165	88	5.7
II:5	F	Het	55–60	35	172	70	45	284	363	12.1
III:4	M	Het	30–35	22	148	83	52	66	89	N/A
III:1	M	Het	25–30	26	209	132	41	180	217	7.4
III:3	F	Het	25–30	48	184	127	45	61	80	N/A
III:2	F	Het	25–30	32	193	119	41	164	90	5.6
III:9	M	Het	20–25	24	157	87	48	111	89	N/A
IV:2	F	Het	5–10	17 (1.0)	N/A	N/A	N/A	N/A	116	N/A
III:8	M	WT	25–30	33	244	169	35	202	82	N/A
III:10	F	WT	20–25	20	N/A	N/A	N/A	N/A	74	N/A
III:7	M	WT	20–25	35	179	114	49	82	94	N/A
IV:3	M	WT	1–5	17	N/A	N/A	N/A	N/A	94	N/A
IV:4	F	WT	1–5	18	N/A	N/A	N/A	N/A	81	N/A

*Hom* homozygous, *Het* heterozygous, *WT* wild type, *F* female, *M* Male. Age adjusted Z scores of BMI values for Het / Hom children (compared to WHO norms) given in parenthesis. N/A not available. Ages (years) given in ranges to obscure patient identity

Homozygotes for the p.E42* *MC4R* mutation exhibited average BMI of 44.83 (44.83 ± 9.7 SD), indicative of extreme obesity (obesity class III) and significantly higher than the BMI of both heterozygous and wild type family members (*p* < 0.005). Individuals heterozygous for the mutation had an average BMI of 29.22 (29.22 ± 10.2 SD), within the upper limits of BMI values defined as overweight. Average BMI of family members homozygous for the wild type *MC4R* allele was 24.46 (24.46 ± 8.98 SD), within BMI normal values.

Fasting blood triglycerides, cholesterol, LDL, HDL, glucose and HbA1C levels were measured for most individuals studied. As seen in Table 2, only the eldest of the 4 homozygous individuals (III:5, Fig. 1a, age 30–35 years) had extremely high levels of cholesterol, triglycerides, LDL, glucose and HbA1C, while the other 3 (ages 5–10 and 25–30 years) had normal values. Similarly, of the 7 heterozygotes, high levels of triglycerides, glucose and HbA1C were found only in the eldest individual (age range 55–60 years, versus age ranges 5–10, 20–25, 25–30, 25–30, 25–30, 30–35 of heterozygotes with normal levels).

## Discussion

*MC4R* mutations are the most common known genetic cause of obesity, affecting 2–3% of the population in various cohorts tested [16, 29]. To date, about 200 *MC4R* genetic variants have been identified, including at least 122 missense mutations, 2 in-frame deletion mutations, 7 nonsense mutations and dozens of frameshift mutations [3, 30], altogether affecting more than 30% of the receptor coding sequence. While it has been suggested that obesity due to *MC4R* mutations can be caused by either haplo-insufficiency or dominant negative activity exerted by the mutant receptor, co-transfection studies show that the extreme majority of mutations analyzed do not have dominant negative activity [3, 15, 31, 32]. Therefore, it is suggested that haplo-insufficiency is the main route through which these mutations exert their effect. While loss-of-function mutations in *MC4R* cause familial forms of obesity, two rare gain-of-function *MC4R* polymorphisms have been identified that are associated with protection against obesity [19]. In fact, we show that in our cohort of 120 control Bedouin whole exome sequences, 4 individuals are heterozygous for one of these variants, namely c.307G > A, p.V103I. Meta analysis of previously published data showed that this gain-of function mutation has a modest negative association with obesity [19]. It is of interest that the prevalence of the p.V103I variant in the Israeli Bedouin community seems to be higher than worldwide (ExAC frequency 0.01743). Notably, another gain-of-function c.751A > C p.I251L *MC4R* variant, that is more clearly negatively associated with obesity worldwide [19], was not found in our Bedouin cohort. Obviously, larger cohorts within this large inbred Bedouin community [33] should be tested to validate statistical significance of these observations.

In families with *MC4R*-associated obesity, obesity tends to have an autosomal dominant mode of transmission, but the penetrance of the disease can be incomplete and the clinical expression variable (moderate to severe obesity), underscoring the role of the environment and other possible modulating genetic factors [34, 35]. As heterozygous *MC4R* mutation carriers are obese, yet present with partial penetrance of the mutations, O’Rahilly and colleagues concluded that the mode of inheritance in MC4R deficiency is codominance with modulation of expressivity and penetrance of the phenotype [36]. It has been suggested that the varying onset and severity of obesity in heterozygous *MC4R* mutation carriers are related to the severity of the functional effects of the mutations. In fact, with many human *MC4R* mutations identified, several research groups (Tao et al. [3], MacKenzie [29], Vaisse et al. [14], Farooqi et al. [15]) classified the *MC4R* mutations based on possible functional consequences: mutations that cause intracellular-retaining of the receptor, defective expression, defective binding, defects in both basal and ligand-induced signaling, etc. However, as only a minority of the variants underwent in-depth functional analysis, validity of such classifications in the context of clinical phenotypic association awaits further studies.

Homozygous or compound heterozygous carriers of *MC4R* mutations are rare [15, 22, 23]. Only few families have been described to date in which multiple heterozygotes and homozygotes of the same mutation are found. We now identified a novel *MC4R* truncation mutation, putatively abolishing all 7 transmembrane domains of the molecule (Fig. 1c). Previous studies reported phenotypic variation in consequences of heterozygous *MC4R* deletion mutations [19, 20]. As the cohort we studied is small, while the average BMI in heterozygous individuals was higher than in wild type family members (29.22 versus 24.24.46, respectively), this difference was not statistically significant, neither in adults nor in children.

Unique to our study, we delineated the mutation-related phenotype in large consanguineous kindred with 4 homozygous and 7 heterozygous individuals, as well as 5 wild type family members. This unique kindred, of few identified thus far, allows insights as to phenotypic effects in heterozygotes versus homozygotes of the same mutation. As evident in Table 2, although the cohort is too small to establish statistical significance, the data clearly point to early-onset obesity in individuals homozygous for the *MC4R* mutation: of the children (ages 1–7 years) within the kindred, the two homozygotes were morbidly obese (BMI 37 and 37; Z scores 3.12, 3.08) as compared to the two wild type individuals (BMI 18 and 17) and the heterozygous individual (BMI 17) that were within normal BMI values (85th BMI-per age percentile). In fact, in spite of the fact that a single kindred is described, the clear morbid obesity (average BMI 44.83) in homozygous individuals is statistically significant compared to the overweight (average BMI 29.22) in heterozygous individuals and the higher end of normal weight (average BMI 24.46) seen in wild type family members. This is in line with previous reports, showing a dramatically more consistent and severe obesity phenotype in homozygotes for *MC4R* mutations than in heterozygotes [16]. Furthermore, the obesity in the homozygotes is of early onset, with BMI Z scores ~ 3 in individuals ages 5–10 years. While previous studies of *MC4R* heterozygotes have shown age-dependent differences in expressivity and a stronger effect in females [16, 18–21, 34], the cohort in the present study is too small to reach conclusions in this regard. Interestingly, although all affected individuals share the same mutation and reside in the same environment (practically the same household), there is variability in phenotypic expression, in the heterozygous individuals in particular, suggesting possible effects of modifier genes. In fact, future studies of such kindreds might be conducive to elucidation of such modifiers.

Previous reports of homozygotes vs heterozygotes of the same *MC4R* mutations did not systematically describe related blood biochemistry values. Fasting blood triglycerides, cholesterol, LDL, HDL, glucose and HbA1C levels were measured for most individuals in our studied kindred. As seen in Table 2, only the eldest of the 4 homozygous individuals (III:5, Fig. 1a, age 30–35 years) had extremely high levels of cholesterol, triglycerides, LDL, glucose and HbA1C, while the other 3 (ages 5–10 and 25–30 years) had normal values. Similarly, of the 7 heterozygotes, high levels of triglycerides, glucose and HbA1C were found only in the eldest individual (age range 55–60 years vs age ranges 5–10, 20–25, 25–30, 25–30, 25–30, 30–35).

## Conclusions

Through studies of a large inbred kindred we demonstrate a novel *MC4R* mutation, practically eliminating all functional domains of the encoded protein. We show that the phenotype in homozygotes for the mutation is significantly more severe than that of heterozygous carriers of the same mutation. While obesity, as delineated through BMI measurements, is evident in homozygotes (yet not necessarily in heterozygotes) at early ages, the metabolic consequences of *MC4R*-related obesity appear in both heterozygotes and homozygotes only at later ages.

## Abbreviations

BMI: Body mass index
LEP: Leptin
LEPR: Leptin receptor
MC4R: Melanocortin-4 receptor
PC1: Prohormone convertase 1
PCR: Polymerase chain reaction
POMC: Proopiomelanocortin
